# Hybrid Fibers with Subwavelength-Scale Liquid Core for Highly Sensitive Sensing and Enhanced Nonlinearity

**DOI:** 10.3390/mi15081024

**Published:** 2024-08-11

**Authors:** Caoyuan Wang, Ruowei Yu, Yucheng Ye, Cong Xiong, Muhammad Hanif Ahmed Khan Khushik, Limin Xiao

**Affiliations:** 1 Advanced Fiber Devices and Systems Group, Key Laboratory of Micro and Nano Photonic Structures (MoE), Key Laboratory for Information Science of Electromagnetic Waves (MoE), Shanghai Engineering Research Center of Ultra-Precision Optical Manufacturing, School of Information Science and Technology, Fudan University, Shanghai 200433, China; caoyuanwang@fudan.edu.cn (C.W.); rwyu20@fudan.edu.cn (R.Y.); 16210720008@fudan.edu.cn (Y.Y.); 21110720019@m.fudan.edu.cn (C.X.); hanif.khushk@bbsutsd.edu.pk (M.H.A.K.K.); 2 Yiwu Research Institute of Fudan University, Chengbei Road, Yiwu 322000, China

**Keywords:** subwavelength waveguide, optofluidics, sensing, nonlinearity

## Abstract

Interest grows in designing silicon-on-insulator slot waveguides to trap optical fields in subwavelength-scale slots and developing their optofluidic devices. However, it is worth noting that the inherent limitations of the waveguide structures may result in high optical losses and short optical paths, which challenge the device’s performance in optofluidics. Incorporating the planar silicon-based slot waveguide concept into a silica-based hollow-core fiber can provide a perfect solution to realize an efficient optofluidic waveguide. Here, we propose a subwavelength-scale liquid-core hybrid fiber (LCHF), where the core is filled with carbon disulfide and surrounded by a silicon ring in a silica background. The waveguide properties and the Stimulated Raman Scattering (SRS) effect in the LCHF are investigated. The fraction of power inside the core of 56.3% allows for improved sensitivity in optical sensing, while the modal Raman gain of 23.60 m^−1^·W^−1^ is two times larger than that generated around a nanofiber with the interaction between the evanescent optical field and the surrounding Raman media benzene-methanol, which enables a significant low-threshold SRS effect. Moreover, this in-fiber structure features compactness, robustness, flexibility, ease of implementation in both trace sample consumption and reasonable liquid filling duration, as well as compatibility with optical fiber systems. The detailed analyses of the properties and utilizations of the LCHF suggest a promising in-fiber optofluidic platform, which provides a novel insight into optofluidic devices, optical sensing, nonlinear optics, etc.

## 1. Introduction

The advancement of optoelectronics has relied on a continued effort to design and fabricate elegant optical waveguides. A fascinating example is the development of the planar silicon-on-insulator slot waveguide that consists of a low-index subwavelength-scale slot embedded by two high-index strips [1], with a significantly enhanced optical confinement in the slot due to the continuity condition of the normal electric displacement at the high-index contrast interface [2]. This ability has attracted intense interest in numerous applications such as optical modulators [3], microring resonators [1], electric pump illuminators [4], all-optical logic gates [5], directional couplers [6], beam splitters [7], polarization combiner [8], power combiner [9], etc. Furthermore, the potential of slot waveguides in optofluidics has been exploited by filling the subwavelength-scale air slots with fluidic samples [10,11]. With stronger optical confinement and higher intensity, these slots can act as efficient light–matter interaction platforms. For example, an approach to optofluidic trapping and transporting nanoparticles and biomolecules using the subwavelength-scale liquid-core slot waveguide has been demonstrated [11]. However, the performance of the planer slot waveguide device in optofluidics may be limited by the high transmission loss induced by the surface roughness [12], the high coupling loss at the interface [13], and the short light–fluid interaction length [11].

One solution to extend the optofluidic capability of slot waveguide devices is to use the in-fiber waveguide geometries. Optical fibers, which can provide low loss, flexibility, robustness, and compactness, can be designed and fabricated to guide light and fluid simultaneously in subwavelength-scale channels over macroscopic interaction lengths and, thus, can significantly enhance the optofluidic interactions. Wiederhecker et al. have proposed a subwavelength-scale air slot of 110 nm in the core region in a silica-based microstructured optical fiber (MOF), creating a new fashion for the light–matter interaction in optical fibers [14]. Nevertheless, the confinement of light in the bore was relatively weak due to the passable refractive index contrast between silica and air. An alternative to enhance light confinement is the use of high-index soft glasses as the background material. As expected, owing to the availability of soft glasses with low softening temperature, they have been used to fabricate MOFs [15,16] with intensity enhancement [17], ultra-high nonlinearity [18], and strong evanescent field for sensing applications [19]. Despite this, when liquid materials instead of air are introduced into the hollow central channel, the fiber still suffers from insufficient index contrast.

Silicon is currently one of the most popular semiconductor materials for optoelectronic applications thanks to its high refractive index, large nonlinearity, wide transparency window, and high damage threshold [20]. Thus, silicon-core optical fibers have been studied for an all-optical switch [21], soliton-like propagation [22], fiber sensing [23], etc. New possibilities arise for improving the local enhancement of light in silicon fiber waveguides, which also allows for wide tailoring of the waveguiding characteristics and nonlinear applications. However, no studies on the design, fabrication, and application of an in-fiber silicon-based slot waveguide have been reported to date.

In this paper, we present a novel subwavelength-scale liquid-core hybrid fiber (LCHF), which incorporates the planar slot waveguide concept into a hollow-core fiber (HCF). The LCHF is a three-layer structure composed of a central hollow core, a silicon layer, and a silica outer cladding. To realize an efficient Stimulated Raman Scattering (SRS) effect, carbon disulfide is selected to infiltrate the slot due to its large nonlinearity. Based on this assumption, the slot waveguide properties of the guided mode in the LCHF are investigated in terms of the mode field distribution and the fraction of power in the core. Then, the Raman effect is studied by revealing the SRS scheme in this fiber structure, optimizing the fiber parameters for efficient SRS, and exploiting the evolutions of the pump and the Stokes powers along the LCHF. The high fraction of power inside the core improves the sensing sensitivity, while the high modal Raman gain indicates a significant low-threshold Raman effect. Moreover, this in-fiber structure provides a platform with compactness, trace sample consumption, reasonable filling time, and an efficient coupling interface. The exquisite design guidance for the utilization of the LCHF offers a fascinating perspective on the development of lab-in-fiber devices, optofluidics, optical sensing, nonlinear optics, etc.

## 2. Structure and Fabrication of the LCHF

**Figure 1:**
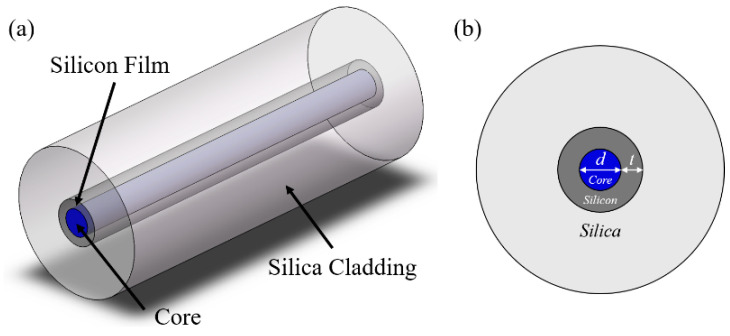
Schematics of the proposed LCHF in (**a**) 3D view and (**b**) cross-section view.

The proposed LCHF is an incorporation of the planar slot waveguide concept into an HCF, which not only has the superiority of planar slot waveguide with a significantly enhanced optical confinement in the subwavelength-scale region but also maintains the natural merits of fiber structure such as robustness, flexibility, and long waveguide lengths. As shown in Figure 1, the LCHF is composed of a subwavelength-scale liquid core encircled by an annular high-index silicon layer within a fused silica cladding.

The fabrication of the LCHF can be achieved with a tapered silica HCF template infiltrated with materials via a high-pressure chemical vapor deposition (HPCVD) method. The HCF is first smoothly down-tapered to prepare a long subwavelength-scale core in its waist region. Then the hollow-core capillary acts as the chemical reaction chamber, and the silicon film can be deposited onto the capillary walls through the control over the deposition conditions such as the temperature and the pressure, where the high pressure can be sustained because of the very high mechanical strength of silica-based optical fibers favors to overcome the mass transport constraints to flow the chemical precursors and enables a strikingly uniform, dense, and conformal annular deposition along the total fiber length [20]. The thickness of the silicon layer *t* and the diameter of the core *d* can be determined by the deposition time. Subsequently, various fluidics can be introduced into the hollow core by modifying the chemical properties of the inner surface, and the produced LCHF can be used to implement the optofluidic experiment.

The structure has fascinating merits over its optical properties and practical utilizations. As an optofluidic element, this structure is advantageous for trace sample consumption and improved sensing sensitivity through a long fiber length. For nonlinear applications, the high refractive index contrast between the core and the annular layer allows for tight mode confinement in the subwavelength-scale core, which facilitates the local enhancement of nonlinear effects. Moreover, due to the high refractive index contrast between silicon and silica, light can be confined effectively in the silicon ring and the liquid-core region, with only a few microns penetration depth into the silica cladding. Furthermore, for practical utilizations, since the working section in the fiber waist region is connected to two untreated sections through smooth transitions, the fiber structure can support adiabatic transmission and adequate mechanical handling, where the cladding diameter of the initial untreated sections is recommended as 125 microns for mechanically splicing to the conventional fiber with good compactness and robustness. All of these advantages in the LCHF show its potential as a meaningful and powerful optofluidic platform for further investigation.

## 3. Waveguide Properties of the LCHF

### 3.1. Basic Assumptions for Simulation

Detailed studies on the LCHFs are conducted using a full vector finite element method (FEM), consisting of the waveguide properties and the Raman characteristics. In our simulation, carbon disulfide is applied as the core material due to its large nonlinearity and its widespread use in studies of fiber-optic Raman effects [10,24,25]. The optical wavelength is chosen as 1553 nm, which is a typical pump wavelength for carbon disulfide to activate the SRS [10]. At 1553 nm, the refractive indices of carbon disulfide (CS_2_), silicon (Si), and silica (SiO_2_) are 1.588, 3.475, and 1.444, respectively [26,27]. A quarter of the cross-section in the LCHF model is used to simplify the calculation process since the structure is symmetric [28]. It should be noted that the polarization orientations of the guided modes should be controlled consistently to support the calculation and the analyses of modal coupling properties, which can be determined by the same boundary condition of a perfect electric or magnetic conductor (PEC/PMC) condition along each symmetric plane [28]. Here, we apply the PEC condition along the horizontal boundary and the PMC condition along the vertical boundary, and, thus, the polarization can be preserved in the vertical direction [28] (defined in the *y*-direction, so the orthogonal direction is defined in the *x*-direction, correspondingly). Despite the fact that we only illustrate the *y*-polarization of the fundamental mode, the circular symmetry in the LCHF ensures the same effect for any polarization direction [14]. Based on these assumptions, the simulations are then conducted with different structural parameters to investigate the optical properties and find optimal geometries as efficient optofluidic platforms for both sensing and SRS applications.

### 3.2. Modal Intensity Distribution in the LCHF

**Figure 2:**
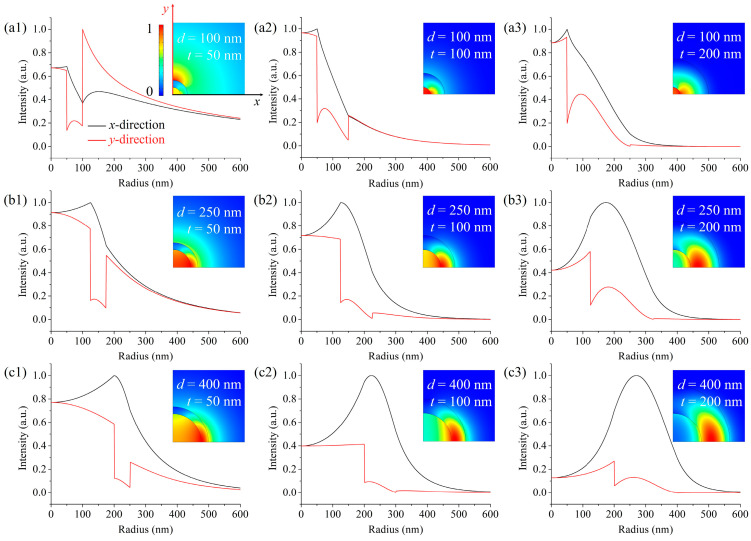
Evolution of normalized modal intensity distributions of LCHFs with the core diameter *d* and the silicon layer thickness *t* of: core diameter = 100 nm, (**a1**) *t* = 50 nm, (**a2**) *t* = 100 nm, (**a3**) *t* = 200 nm; core diameter = 250 nm, (**b1**) *t* = 50 nm, (**b2**) *t* = 100 nm, (**b3**) *t* = 200 nm; core diameter = 400 nm, (**c1**) *t* = 50 nm, (**c2**) *t* = 100 nm, (**c3**) *t* = 200 nm. Insets correspond to a quarter of the mode patterns for fundamental modes (HE_11_ modes, polarized along the *y*-direction).

The waveguide properties of the proposed subwavelength-scale LCHF are first studied in terms of the modal intensity distribution. Figure 2 shows the evolution of the normalized intensity distributions of LCHFs with the core diameter *d* and the silicon layer thickness *t*, and the insets are corresponding mode patterns for the fundamental modes (HE_11_ modes, polarized along the *y*-direction). These subwavelength-scale mode patterns are no longer Gaussian-like, and a tighter confinement of light inside the core appears when *d* is smaller. Here, we detail the distribution in two orthogonal lines that run through the center (as shown in Figure 2(a1)) to describe the variation in optical intensity. Figure 2(a1–a3) shows the modal intensity distributions of LCHFs with a smaller core diameter *d* of 100 nm. It is noted that a large portion of the mode in Figure 2(a1) spreads into the silica cladding, which is derived from the loose light confinement in the extremely thin two inner layers. In comparison to Figure 2(a1), tighter light confinements can be observed with a larger core (Figure 2(b1)) or a thicker silicon layer (Figure 2(a2)). For the other two cases shown in Figure 2(a2,a3), the higher intensities in the *x*-direction are concentrated near the core/silicon layer interfaces, with asymmetric reductions toward the center and the periphery, respectively. Since the core is sufficiently small, the evanescent field that leaks into the central core decays only a little, and the intensity reduction toward the center is not pronounced. With the increase in *t* from 100 to 200 nm, the intensity toward the center decays more rapidly, which arises from the stronger confinement of light in the high-index silicon layer. Moreover, at the core/silicon layer and silicon layer/silica cladding interfaces, the intensities are continuously varied. In the *y*-direction, the region with higher intensities lies inside the core. The optical intensities are discontinuous at the two interfaces due to the continuity condition of the normal electric displacement in the *y*-polarization direction, with an obvious down-jump at the core/silicon-layer interface and a small up-jump at the silicon layer/silica cladding interface. With the increase in *t*, the discontinuities at the two interfaces are less significant, which can be explained by the larger decays of intensities due to the tighter confinement of light in the high-index layer.

As *d* increases from 100 to 400 nm, the waveguide properties are similar but weaker in the LCHFs compared with those in small-core LCHFs, where the intensities show larger decays into the center in the *x*-direction and slighter jumps at the interfaces in the *y*-direction. Figure 2b,c shows that the intensity distributions for *d* equal 250 nm and 400 nm, respectively. Especially when *d* increases to 400 nm, a smaller portion of intensity decays into the core. Furthermore, with the increase in *t*, the peak intensity moves into the silicon layer, and a weaker intensity spreads into the core of the LCHF. A series of simulations demonstrate the waveguide properties in the designed LCHFs, and a smaller core enables a higher intensity in the core.

### 3.3. Fraction of Power in the LCHF Core

**Figure 3:**
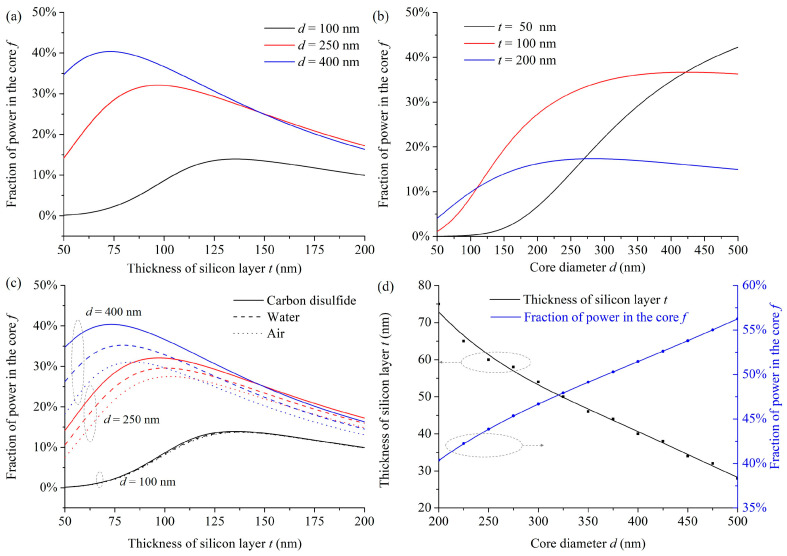
(**a**,**b**) Variations in the fraction of power in the core *f* in the LCHF with different parameters. (**c**) Variations in the fraction of power in the core *f* when the core is filled with different liquids, with different thicknesses of silicon layer *t*. (**d**) Variations in the optimal silicon layer thickness *t*, and the corresponding peak power fraction *f,* with the core diameter *d*.

The confinement of the guided fundamental mode in the designed LCHF is then quantified by calculating the fraction of power in the core. The efficiency of the light–matter interaction in an LCHF highly depends on the fraction of power in the core *f* that is defined by [29]

(1) $$f=\frac{\int_{core}\left(E_{x}H_{y}-H_{x}E_{y}\right)dxdy}{\int_{total}\left(E_{x}H_{y}-H_{x}E_{y}\right)dxdy}$$

where *E_x_*, *E_y_* and *H_x_*, *H_y_* are the transverse electric and magnetic fields of the mode, respectively. Figure 3a shows the variations in the calculated fraction of power in the core *f* in the LCHF with the silicon layer thickness *t*. For a fixed core diameter *d*, an optimal *t* can be observed with a maximum *f*, where the left increase in *f* in the curve results from the reduced leakage of mode in the outer silica cladding, while the right decrease in *f* is from the enhanced confinement of light in the thicker silicon layer (see the evolution of mode patterns in Figure 2). Moreover, a thinner silicon layer can achieve a higher optimal power fraction in the core when the core diameter is larger because the mode guidance depends on the cooperative effect of the inner two layers. In detail, the power fraction in the core is based on a global consideration of the portion of inner layers in the whole fiber and the portion of the core in the inner layers. For the three core diameters of 100 nm, 250 nm, and 400 nm, the calculated optimal power fractions in the core at the corresponding silicon layer thicknesses are 14.0% at 135 nm, 32.1% at 95 nm, and 40.4% at 75 nm, respectively.

The variations in the calculated fraction of power in the core *f* in the LCHF with the core diameter *d* are presented in Figure 3b. At these smaller core diameters, the power fractions in the core are smaller for LCHFs with thin silicon layers. However, it performs a fast increase and finally achieves a large value. For example, when *d* ranges from 50 to 500 nm, *f* increases from 2.1% to 42.2% when *t* equals 50 nm. For the other two cases, the smaller *f* occurs at a smaller *d* with a larger *t*. This agrees well with the analyses in Figure 3a. In detail, the peak power fractions in the core at the corresponding core diameters when *t* equals 100 nm and 200 nm are, respectively, 36.7% at 430 nm and 17.4% at 290 nm. The results indicate that an LCHF with a larger core and thin silicon layer can maximize the power fraction in the core and, thus, promote the interaction between the confined light and fluid.

As comparisons, the mode confinement properties in the LCHF structures with other liquid materials are characterized. Figure 3c shows the variation in the calculated power fraction in the core *f* when the core is filled with carbon disulfide, water, and air, respectively, with the silicon layer thickness *t*. The refractive indices of water and air at the wavelength of 1553 nm are set as 1.316 [30] and 1, respectively. For the water-core and air-core LCHFs, as expected, the calculated *f* values are always smaller than those in the CS_2_-core LCHFs with the same structural parameters since a higher-index core material favors stronger mode confinement in the inner layers. Only slight differences in *f* among the LCHFs with different core materials are noticed when *d* is small, for example, when *d* equals 100 nm since the core is too small to act on the mode guidance. With the increase in *d* and the reduction in *t*, these differences become more apparent, demonstrating that the higher-index CS_2_-filled core can decrease the effective index contrast between the inner two layers and, thus, can increase the leakage of mode to the core. For the LCHFs with *d* of 400 nm, the peak *f* values in the water-core and air-core LCHFs are 35.5% and 31.3%, with 4.9% and 9.1% reductions compared with that in the CS_2_-core LCHF, respectively. The results indicate that the high-index CS_2_-filled core facilitates confining the fundamental mode in the core, especially for cases with large cores and thin silicon layers, where the high fractions of power in the cores allow for enhanced light–fluidic interactions.

The simulations in Figure 3a–c indicate that there is always an optimal silicon layer thickness for a given core diameter to realize the highest fraction of power in the core. Figure 3d shows the variations in the optimal silicon layer thickness *t* and the peak power fraction *f* with the core diameter *d*. When *d* increases from 200 nm to 500 nm, the optimal *t* decreases from 75 nm to 28 nm, and *f* increases from 40.4% to 56.3%. The results exhibit the great superiority of the designed LCHFs with a larger core and a thinner silicon layer in improving the efficiency of optofluidics. When applying a 10-cm-long LCHF with the maximum power fraction in the core as the sensing element, i.e., the core diameter of 500 nm and the silicon layer thickness of 28 nm, the infiltrated liquid volume is 19.63 pL. The trace sample consumption indicates the potential for practical construction of the sensing platform.

## 4. Modeling of Raman Effect in the LCHF

### 4.1. Scheme on Raman Effect in the LCHF

The generation of the Raman effect in the LCHF is first introduced. When the light propagates in an LCHF, a significant portion of the HE_11_ mode enters the core region and interacts with the infiltrated Raman media, carbon disulfide. The Raman effect starts with the Spontaneous Raman Scattering process, where the incident pump photons are scattered and then form other photons with lower energy through the interactions with molecular vibrations [31]. Some of these scattered photons are guided by the LCHF and then can be amplified by expensing the initial pump beam through the stimulated Raman amplification [31]. Thus, a large part of the initial pump beam is converted into the Stokes beam with a longer wavelength [31], and finally, both of the two beams can propagate along the LCHF.

**Figure 4:**
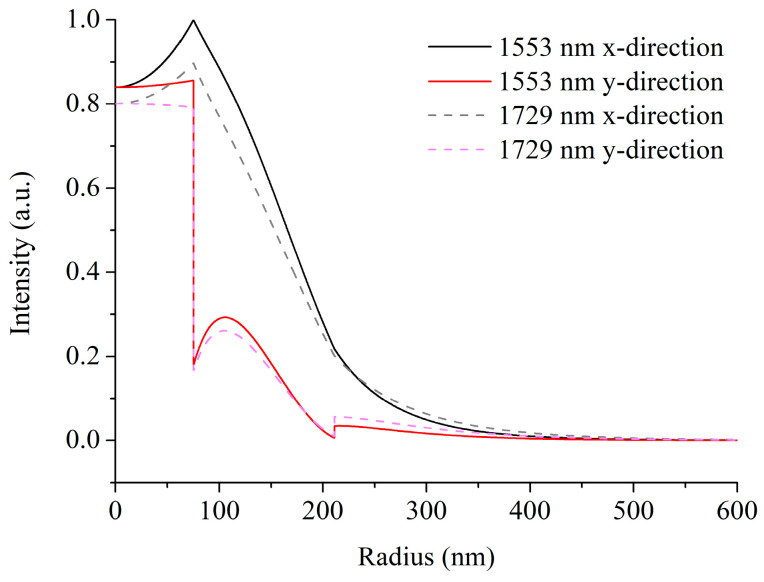
Normalized transverse intensity profiles of the HE_11_ pump mode and the HE_11_ first-order Stokes mode in two orthogonal directions for a CS_2_-core LCHF structure with *d* of 150 nm and *t* of 136 nm.

The transverse intensity profile of the Stokes beam is then investigated. For carbon disulfide, when the wavelength of the pump beam is 1553 nm, the first-order Stokes beam is at 1729 nm [10]. Figure 4 shows the transverse intensity profiles of the HE_11_ pump mode and the HE_11_ first-order Stokes mode in two orthogonal directions for a CS_2_-core LCHF structure with a core diameter of 150 nm and a silicon layer thickness of 136 nm. It can be seen that the modal intensity profiles of the pump beam and the first-order Stokes beam are similar but do not entirely overlap. In detail, the first-order Stokes mode has lower intensities in the inner two layers and higher intensities in the outer cladding compared with those of the pump mode, and the fractions of power in the core are 21.01% for the pump beam and 19.56% for the first-order Stokes beam. The imperfect overlap indicates the existence of other Stokes modes excited by other pump modes. However, only the HE_11_ pump mode is analyzed in our study since the attenuation of other modes is much larger than that of the Raman gain [31].

The SRS effect in the designed LCHFs can be measured by the coupled-mode equations, with the coupling between the pump beam and the generated Stokes beam polarizing along the same direction [32]. Here, we consider the modal Raman gain *g_s_* developed by the vectorial-based nonlinear Schrödinger equation to analyze the first-order Raman effect, which considers the significant longitudinal components of the electric field vector for the subwavelength-scale optical fiber structure with a high refractive index contrast and the differences in the modal field distributions of the pump and Stokes fields. The modal Raman gain can be calculated by [32]

(2) $$g_{s}=\frac{\varepsilon_{0}^{2}c^{2}\iint_{active}g_{R}n^{2}{\left|E_{p}\cdot E_{s}^{*}\right|}^{2}\;dA}{\left(\iint_{total}(E_{p}^{*}\times H_{p})\cdot \hat{z}\;dA\right)\left(\iint_{total}(E_{s}^{*}\times H_{s})\cdot \hat{z}\;dA\right)}$$

where *ε_0_* is the vacuum dielectric constant; *c* is the speed of light in vacuum, and *n* is the refractive index of the Raman medium. *g_R_* is the Raman gain coefficient for the Raman media, expressed in m·W^−1^, with different values at the Stokes wavelength for the material. ***E*** and ***H*** represent the vectorial electric and magnetic fields, and the subscripts *p* and *s* stand for the pump beam and the Stokes beam, respectively. The calculated *g_s_* is the modal Raman gain, expressed in m^−1^·W^−1^. For the first-order SRS effect, the Raman gain coefficient *g_R_* is 2.7 × 10^−11^ m·W^−1^, and the line width of the Raman spectrum is 0.7 cm^−1^ [10,33]. The refractive index for carbon disulfide at the wavelength of 1729 nm is adopted as 1.588 [26]. Based on this frame, in the next section, we will optimize the fiber structure to realize efficient SRS effects.

### 4.2. Optimization for Efficient SRS in the LCHF

**Figure 5:**
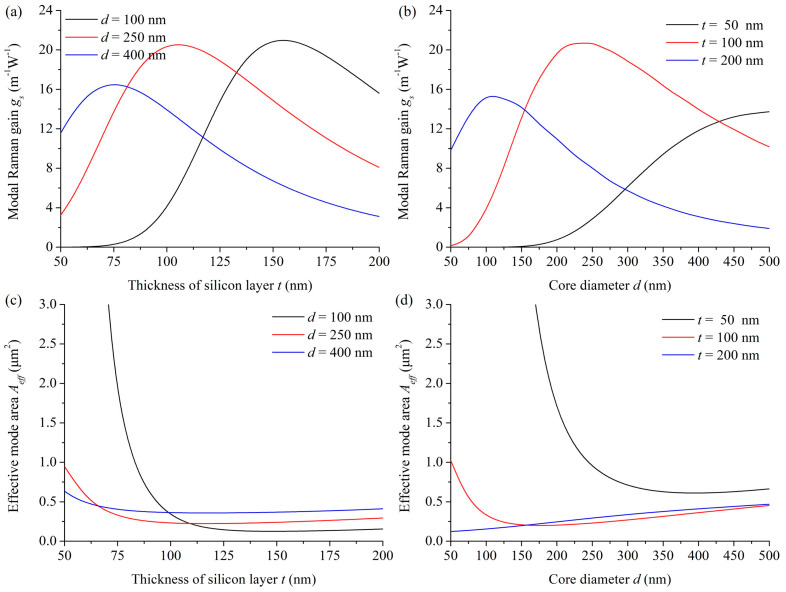
Variations in the modal Raman gain *g_s_* with (**a**) different core diameters of 100 nm, 250 nm, and 400 nm as functions of the thickness of silicon layer, (**b**) different thicknesses of 50 nm, 100 nm, and 200 nm as functions of the core diameter. Variations in the effective mode area *A_eff_*, with (**c**) different core diameters of 100 nm, 250 nm, and 400 nm as functions of the thickness of silicon layer, (**d**) different thicknesses of 50 nm, 100 nm, and 200 nm as functions of the core diameter.

The optimization of the SRS effect in the LCHF is guided by the modal Raman gain, and the influence of the structural parameters for an LCHF on the generated SRS effect is first studied. Figure 5a,b shows the variations in the modal Raman gain *g_s,_* with the silicon layer thickness *t* and the core diameter *d*, respectively. In Figure 5a, for a fixed *d*, *g_s_* increases to the peak and then decreases as *t* increases. The peak modal Raman gains at the corresponding silicon layer thicknesses are 21.17 m^−1^·W^−1^ at 150 nm, 20.71 m^−1^·W^−1^ at 100 nm, and 16.80 m^−1^·W^−1^ at 75 nm for the core diameters of 100 nm, 250 nm, and 400 nm, respectively. For Figure 5b, *t* is fixed, *g_s_* first increases to the peak, and then it begins to fall gradually when *d* increases. And the peak modal Raman gains at the corresponding core diameters are 13.73 m^−1^·W^−1^ at 500 nm, 20.71 m^−1^·W^−1^ at 250 nm, and 15.58 m^−1^·W^−1^ at 100 nm for the silicon layer thicknesses of 50 nm, 100 nm, and 200 nm, respectively. These variation trends and peak locations agree well with the evolutions of power fraction in the core that have been mentioned in part 3.3, as a high power fraction in the core is beneficial for enhancing the interaction between the guided core mode and the Raman media and, thus, improving the modal Raman gain. However, a smaller core and a correspondingly thicker silicon layer can achieve a larger peak modal Raman gain. It is related to the reduced mode area and, thus, the enhanced power density. Consequently, we consider explaining these variations in *g_s_* with the combination effect induced by the effective mode area and the overlap between the guided mode field and the Raman-active core, i.e., the fraction of power in the core.

To verify this idea, we calculated the modified effective mode area *A_eff_* in the vectorial model that was redefined as follows [32]:

(3) $$A_{eff}=\frac{\left(\iint_{total}(E_{p}^{*}\times H_{p})\cdot \hat{z}\;dA\right)\left(\iint_{total}(E_{s}^{*}\times H_{s})\cdot \hat{z}\;dA\right)}{\iint_{total}(E_{p}^{*}\times H_{p}\cdot \hat{z})(E_{s}^{*}\times H_{s}\cdot \hat{z})\;dA}$$

and put it in Figure 5c,d to construct its relationship with the modal Raman gain. As expected, the variation trend of the effective mode area is opposite to that of modal Raman gain in general. Both curves indicate that *A_eff_* first decreases to a minimum and then increases slightly with the increase in *t* (Figure 5c) or *d* (Figure 5d). In the smaller LCHF structures, as the size of the inner layers increases, the mode is confined more tightly in the center, and thus, *A_eff_* decreases first to a minimum. Then, *A_eff_* rises, following the enlargement of inner layers. For Figure 5c, the calculated minimum effective mode areas at the corresponding silicon layer thicknesses are 0.12 μm^2^ at 150 nm, 0.22 μm^2^ at 120 nm, and 0.36 μm^2^ at 120 nm for the core diameters of 100, 250, and 400 nm, respectively. For Figure 5d, the calculated minimum effective mode areas at the corresponding core diameters are 0.61 μm^2^ at 390 nm, 0.20 μm^2^ at 170 nm, and 0.12 μm^2^ at 50 nm, for the silicon layer thicknesses of 50, 100, and 200 nm, respectively. These ultra-small effective mode areas of less than 1 μm^2^ imply more intense optical fields and higher nonlinearities, which benefits the realization of the efficient SRS effect.

The relationship between the modal Raman gain, the fraction of power in the core, and the effective mode area are noticed in the comparisons of the optimal cases in the figures. Here, the curves of the core diameter *d* equal 250 nm in Figure 3a and Figure 5a,c, for example. The optimal values in modal Raman gain *g_s_* (Figure 5a), fraction of power *f* (Figure 3a), and effective mode area *A_eff_* (Figure 5c) are located at the silicon layer thicknesses *t* of 100, 95, and 120 nm, respectively. As *t* increases from 50 to 100 nm, *g_s_* rises sharply to the peak first due to the decrease in *A_eff_*, showing the significant enhancement in the optical field. Then, *g_s_* decreases when *t* increases further because the field starts to escape to the silicon layer and, thus, the overlap with the Raman-active core is reduced, which is the dominant factor that affects the modal Raman gain since the mode area changes slightly. Thus, it can be verified that the modal Raman gain depends on the combination effect induced by the effective mode area and the overlap between the pump beam and the Stokes media.

**Figure 6:**
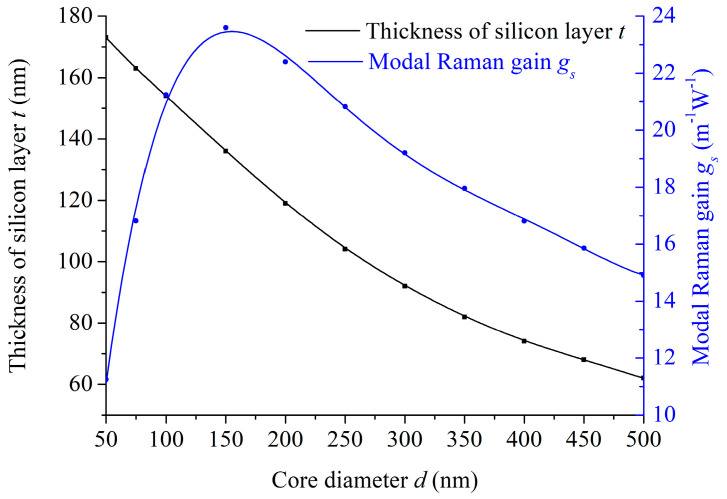
Variations in the optimal silicon layer thickness *t* and the corresponding modal Raman gain *g_s_* with the core diameter *d*.

The simulations in Figure 5 demonstrate that the modal Raman gain in the LCHF can be optimized by changing the silicon layer thickness or the core diameter. Here, each silicon layer thickness corresponds to the optimal modal Raman gain in several fixed core diameters. As the curve in Figure 6 shows, to achieve an optimal SRS effect at each core diameter, the silicon layer thickness *t* reduces as the core diameter *d* increases, owing to the integrated influence of the inner two layers on the modal confinement while the corresponding modal Raman gain *g_s_* rises sharply to the peak and then declines, which indicates a smaller core and a corresponding thicker silicon layer in the LCHF structure can realize the globally optimal modal Raman gain. With the variation in the core diameter from 50 to 500 nm, the calculated optimal modal Raman gain increases from 11.25 m^−1^·W^−1^ to the peak of 23.60 m^−1^·W^−1^ and falls gradually to 14.91 m^−1^·W^−1^, where the peak appears when the core diameter is 150 nm, and the silicon layer thickness is 136 nm. Compared with the reported modal Raman gain of 7.3 m^−1^·W^−1^ that is generated from the interaction of the evanescent field around a nanofiber and the Raman media benzene-methanol [31], our designed fiber structures have significant superiority in enhancing the Raman effect.

### 4.3. Power Evolution Along the LCHF

The evolutions of the pump and the Stokes powers along the LCHF in a continuous-wave (CW) experiment are characterized based on the proposed fiber structure with the highest modal Raman gain. The coupled-wave equations in the quasi-CW regime have provided the variations in the pump power *P_p_* and the first-order Stokes power *P_s_*, with the distance *z* along the fiber [31]:

(4) $$\left\{\begin{array}{c} \frac{dP_{p}}{dz}=-\frac{\omega_{p}}{\omega_{s}}g_{s}P_{p}P_{s}\\ \frac{dP_{s}}{dz}=g_{s}P_{p}P_{s} \end{array}\right.$$

where *ω_p_* and *ω_s_* are the angular frequencies of the pump beam and the Stokes beam, respectively. The initial pump power is determined by the power at the fiber input, while the initial Stokes power can be considered as a net result of the spontaneous emission or an introduction of a seed term that is expressed by [31] as follows:

(5) $$P_{s}(z=0)=\frac{\sqrt{\pi }\hbar \omega_{s}\Delta \nu_{FWHM}}{2\sqrt{\int_{0}^{L}g_{s}P_{p}(z=0)\;dz}}$$

where *ħ* is the reduced Planck constant; *Δν_FWHM_* is the full width at half maximum of the Raman gain curve, and *L* is the length of the LCHF. We consider a uniform CS_2_-filled LCHF section with an axial length of 10 cm, whose core diameter is 150 nm; silicon layer thickness is 136 nm, and the modal Raman gain is 23.60 m^−1^W^−1^. In this case, the initial Stokes power *P_s_*(*z* = 0) is calculated on the order of 10^−8^.

**Figure 7:**
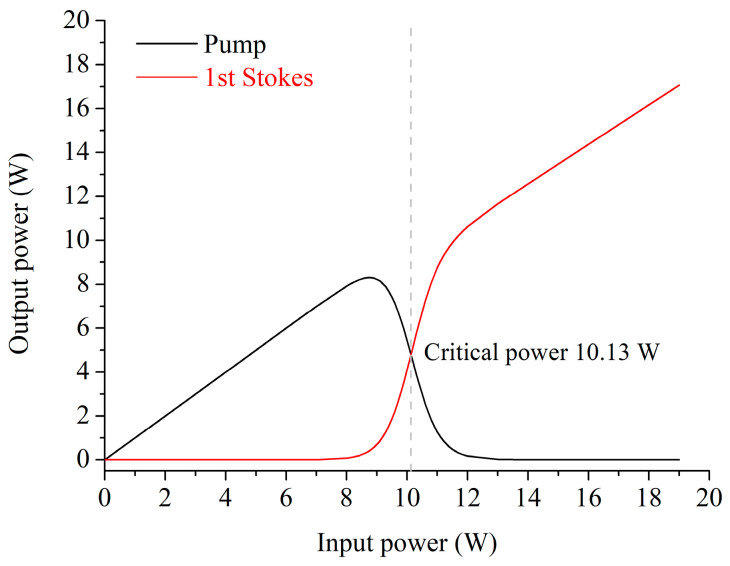
Evolutions of the pump and the first-order Stokes powers, with the input power for the LCHF with the highest modal Raman gain (*d* of 150 nm and *t* of 136 nm) in a CW experiment. The dash line represents the critical power.

Figure 7 shows the calculated power variations in the two beams along the LCHF, with the input power in a CW experiment. When the input power is less than ~8 W, most of the output beam is the 1553 nm pump beam. Then, the 1729-nm first-order Stokes beam increases rapidly with the rise in the input power to ~12 W, and meanwhile, the output pump power decreases dramatically. After that, the pump beam almost totally converted into the Stokes beam. It is worth noting that the critical power for the first Stokes beam *P_crit_* is 10.13 W, where the pump power is equal to that of the first-order Stokes power. The small critical power here is around 21 times lower than that in the previous report based on the evanescent Raman interaction [31], showing great potential in low-threshold Raman applications. The practical utilization of this 10-cm-long LCHF element in SRS applications requires a sample volume of 1.77 pL, which suggests the potential for trace-sample consumption with a highly efficient SRS effect.

## 5. Conclusions

In conclusion, we have proposed a subwavelength-scale LCHF and investigated its waveguide properties and Raman characteristics. The LCHF is a novel design that combines the planar slot waveguide and an HCF, which is composed of a central hollow core infiltrated with carbon disulfide, a silicon layer, and a silica outer cladding. We have simulated the mode field distribution and the fraction of power in the core to study the waveguide properties of the LCHF, where the high fraction of power inside the core of 56.3% allows for sensitive sensing. The Raman effect has been studied by revealing the SRS scheme in this fiber structure, optimizing the fiber parameters for efficient SRS, and exploiting the evolutions of the pump and the Stokes powers along the LCHF. The high modal Raman gain of 23.60 m^−1^·W^−1^ is two times larger than that generated around a nanofiber with the interaction between the evanescent optical field and the surrounding Raman media benzene-methanol, enabling a significant Raman effect with a low threshold. Furthermore, the in-fiber optofluidic platform has trace sample consumption, low-loss optical transmission, and an efficient coupling interface that favors the practical utilization of the device and system. The detailed analyses of the properties and utilizations of the LCHF provide a novel fashion into optofluidic devices, optical sensing, nonlinear optics, etc.

## Data Availability

Data underlying the results presented in this paper may be available from the corresponding author upon reasonable request.
